# PARP Inhibition in Colorectal Cancer—A Comparison of Potential Predictive Biomarkers for Therapy

**DOI:** 10.3390/ph18060905

**Published:** 2025-06-17

**Authors:** Abdulaziz Alfahed

**Affiliations:** Department of Medical Laboratory, College of Applied Medical Sciences, Prince Sattam bin Abdulaziz University, Al-Kharj 11942, Saudi Arabia; a.alfahed@psau.edu.sa

**Keywords:** colorectal cancer, PARP inhibition, predictive biomarkers, large-scale state transition (LST), microsatellite instability (MSI), *PARP1* expression, *PARP2* expression, *TP53* mutation

## Abstract

**Background/Objectives**: PARP inhibitors (PARPis) currently play frontline roles in the management of prostate, pancreatic, ovarian and breast cancers, but their roles in colorectal cancer (CRC) management have yet to be clarified. Importantly, the specific predictive biomarkers for PARPis in CRC are still matters of investigations. The aim of this study is to identify the potential predictive biomarkers of PARP inhibition in CRC. **Methods**: Gene set enrichment analyses (GSEAs) and drug ontology enrichment analyses (DOEAs) of PARPi response gene sets were applied as the surrogates of PARPi response to two CRC cohorts in order to compare the predictive capacities of *TP53* mutation status, MSI status, as well as *PARP1* and *PARP2* expression for PARP inhibition to those of a homologous repair deficiency surrogate, and large-scale state transition (LST). Differential enrichment score (ES) and ontology enrichment (OE) analyses were used to interrogate the differential correlation of the predictive biomarkers with PARPi response, relative to LST. **Results**: The results demonstrated that LST-low, rather than LST-high, CRC subsets exhibited an enrichment of the PARPi response, in contrast to what has been established for other cancers. Furthermore, CRC subsets with wild-type *TP53*, positive MSI, as well as high *PARP1* and *PARP2* expression exhibited an enrichment of the PARPi response gene sets. Moreover, there was no differential enrichment of the PARPi response between LST and each of the MSI statuses, *PARP1* expression and *PARP2* expression. Furthermore, the preliminary differential enrichment observed between the LST-based and *TP53* mutation status-based PARPi responses could not be validated with further testing. **Conclusions**: MSI status, *TP53* mutation status as well as *PARP1* and *PARP2* expression may be substitutes for low LST as predictive biomarkers of PARPi response in CRC.

## 1. Introduction

Colorectal cancer remains an important public health disease worldwide [1], in spite of the effort that has been expended towards understanding its molecular pathogenesis [2]. Currently, CRC is the third commonest cancer and the fourth cancer in terms of highest mortality rates [1]. These dismal statistics warrant continuous efforts at elucidating CRC biology in order to uncover the biomarkers of prognostic and predictive relevance [3,4].

PARP inhibition has become a foremost strategy for cancer therapy in recent times [5,6]. Currently, PARP inhibitors (PARPis) are major players in the first- and later-line therapies for breast, prostate, ovarian and pancreatic cancers [5,7], for which the Food and Drug Administration has approved multiple PARPis for therapies [5]. This study explored the genomic data of CRC cohorts for potential biomarkers of PARP inhibition. Although PARP inhibition has been investigated in CRC cell culture studies [8,9,10], the specific predictive biomarkers for the PARPis in CRC are still a matter of clinical and preclinical investigations. The approved biomarkers for PARPi response prediction in breast, ovarian, pancreatic and prostate cancers are *BRCA* alterations and homologous repair deficiency (HRD) [11,12,13]. And whilst HRD is detectable in CRC using next generation sequencing and DNA SNP array methodologies [8], with the frequency of *BRCA1* and *BRCA2* mutations for CRC in the order of 0.2–2.8%, *BRCA* mutations are not common molecular alterations in this cancer [14,15,16]. Moreover, HRD scores may not fully capture the context in which *PARP1* and *PARP2* protect the CRC genome against worsening genotoxic stress, nor the contexts in which their inhibition would be beneficial for tumor cell killing [8]. Furthermore, HRD detection has demonstrated certain features that limits its clinical usefulness as a dynamic biomarker for responses to PARP inhibition [17]. These limitations include the finding that genomic scars detected by HRD tests persist in spite of the reinstatement of homologous recombination repair function that may occur as part of cancer evolution [17]. The PARPi biomarkers that have been interrogated in CRC, including *TP53* and *ATM* mutation statuses, and microsatellite instability (MSI) [8,10,18]. The aim of this study is to explore the CRC expression datasets with a view to identifying the predictive biomarkers for PARP inhibition in CRC. The specific objectives include the following: (i) to elucidate the biological significances of the PARPi targets, *PARP1/2*, in CRC; (ii) to clarify the roles of HRD in predicting PARPi response in CRC; and (iii) to interrogate the potential predictive relevance of *PARP1/2* expression, *TP53* mutations, *ATM* expression, MSI status, aneuploidy and fraction genome altered (FGA) in PARP inhibition, and compare these findings to the HRD scores and HRD surrogates. The study hypothesis is that the biomarkers *TP53* mutations, MSI, FGA, *ATM* expression and *PARP1/2* expression may be more relevant to CRC biology than HRD because of the rarity of *BRCA* mutations in CRC and hence, may also be suitable markers for PARPi in this cancer type as is the case for HRD and its surrogates.

## 2. Results

PARP1 and PARP2 expression exhibited a direct relationship with each other, suggesting that both PARP genes may function together in the same biological pathways (R = 0.242, *p* < 0.001; X^2^ = 17.663, *p* < 0.001). For the purpose of GSEA, the HRD and LST scores were dichotomized using biologically relevant thresholds from previous studies.

### 2.1. PARP1 and PARP2 Expression Display Correlations with the Clinico-Molecular and Genomic Features of CRC

**Figure 1:**
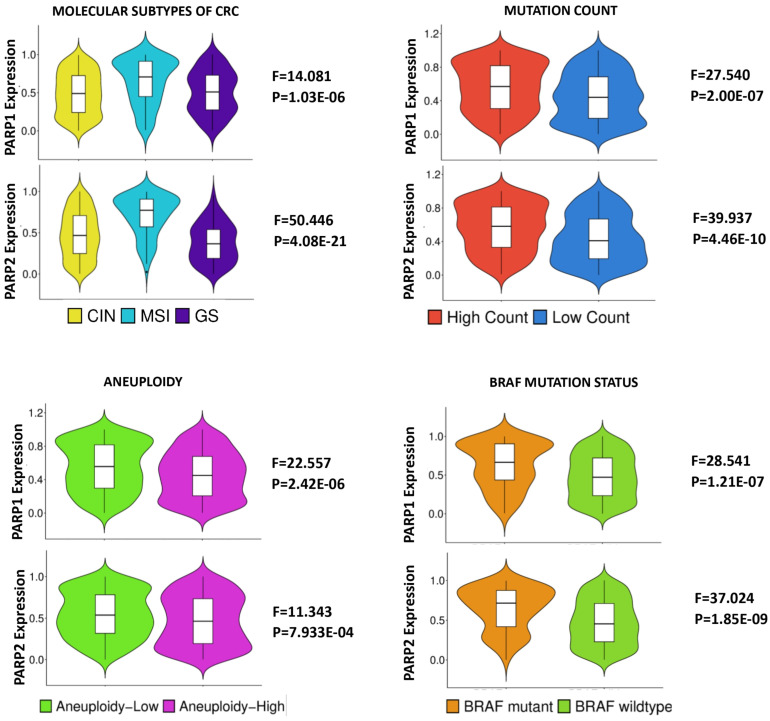
Violin plots showing the correlation of the molecular subtypes, mutation count, Aneuploidy and *BRAF* mutation status with *PARP1* and *PARP2* expression in CRC. Microsatellite instability-positive, low Aneuploidy score, high mutation count and mutant *BRAF* CRC cases have a significantly higher mean expression of *PARP1* and *PARP2*. Sub-figures: **upper panel** = *PARP1* expression; and **lower panel** = *PARP2* expression.

One-way ANOVA was used to test for differences in the PARP1 and PARP2 expression levels between the clinicopathological and molecular subsets of CRC. Both PARP1 and PARP2 expression displayed significant correlations with MSI status, the molecular subtypes of CRC, aneuploidy and BRAF mutations but not TP53 mutations, FGA or LST. Specifically, *PARP1* and *PARP2* expression were higher in MSI more than MSS tumours, and in Hypermutated more than either chromosomal instability (CIN)/Epithelial or genome-stable (GS)/Mesenchymal tumours. Moreover, *PARP1* and *PARP2* expression were lower in tumor subsets with high Aneuploidy score, although the correlation with LST and FGA did not achieve statistical significance for *PARP1* and *PARP2*, respectively (Supplementary Materials S1 and Figure 1). The results confirmed the hypothesis that PARP1 and PARP2 are relevant to the molecular pathogenesis of CRC. Specifically, the results showed that the PARP genes may function in the maintenance of genomic stability in CRC with low chromosomal instability. In addition, PARP1 and PARP2 expression displayed direct correlations with Mutation Count and TMB, highlighting their association with MSI and Hypermutated CRC subsets (Supplementary Materials S1 and Figure 1). Furthermore, high PARP2 expression, but not PARP1, showed an association with right-sided tumors, late pathological tumor stages, and disease-free survival (Supplementary Materials S2 and S3). Also, bivariate correlation demonstrated indirect correlations between the PARP genes and their promoter methylation in the TCGA cohort (methylation data were not available for the Sidra-LUMC cohort), while one-way ANOVA showed that PARP1 and PARP2 copy number alterations (CNAs) were correlated with PARP1 and PARP2 expression, respectively, in both CRC cohorts (Supplementary Materials S4). The results of multiple linear regression analyses showed that both copy number status and methylation states contribute independently to the deregulation of PARP1 expression in CRC, whereas PARP2 CNA, but not methylation, was independently associated with PARP2 expression deregulation. (Supplementary Materials S4). The overall results demonstrated that PARP1 and PARP2 may have biological roles in CRC pathogenesis.

### 2.2. PARPi Response Gene Set Validation in Using Breast Cancer (BC) Cohort

**Figure 2:**
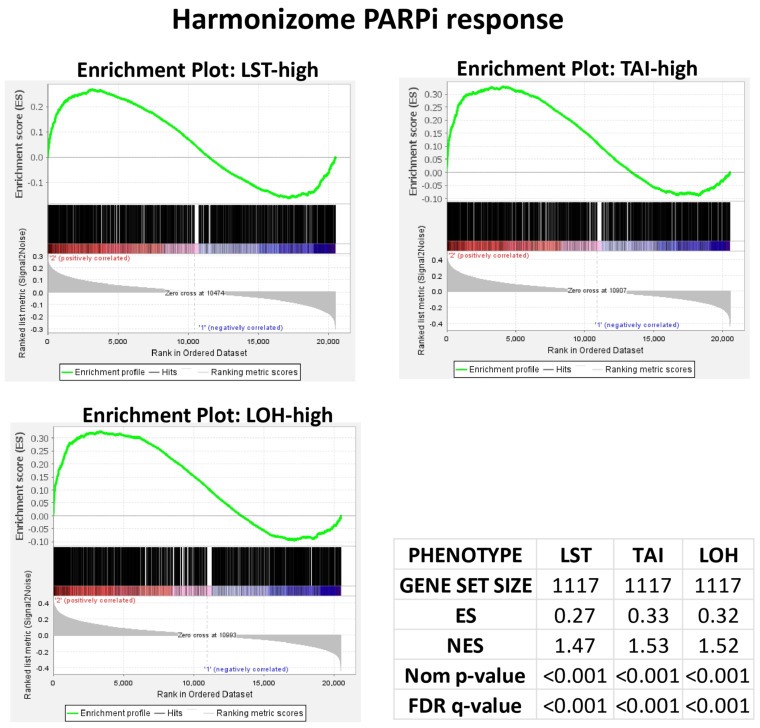
Enrichment plots from the gene set enrichment analyses of the Harmonizome PARPi response gene set in the BC cohort, showing an enrichment of the PARPi response in the high LST, TAI and LOH subsets of BC at nominal *p* values and an FDR of <0.001. The LST-low, TAI-low and LOH-low subsets of BC did not show any enrichment. The results validate the gene set as a surrogate for PARPi response.

To validate the utility of PARPi gene set enrichment and drug ontology enrichment analyses (GSEAs and DOEAs) for PARPi response surrogacy, as well as to select the most sensitive PARPi response gene sets for application to the colorectal (CRC) cohorts, and confirm which HRD state (high versus low) in BC exhibits enrichment for PARPi response, GSEAs were performed with TAI, LOH, LST, and *BRCA1* and *BRCA2* expression phenotypes for the BC cohorts using 22 PARPi gene sets for olaparib, rucaparib, veliparib and talazoparib. The gene sets were obtained from the Harmonizome database (https://maayanlab.cloud/Harmonizome/; accessed on 28 April 2025) and compiled into a single PARPi response gene set of 1242 genes. The results demonstrated an enrichment of the PARPi response gene set in the HRD-high, TAI-high, LST-high, LOH-high and BC subsets, thereby validating the GSEAs as surrogates for PARPi response (Figure 2). The results also demonstrated that the high HRD/HRD surrogate subset of BC were enriched in the PARPi response, in keeping with the established relationship between HRD and PARPi responses in BC. The enriched genes from the BC analyses were compiled into a PARPi response gene set for the CRC cohort analyses.

### 2.3. PARPi Response Enrichment in CRC Cases with Low Chromosomal Instability (CIN)

**Figure 3:**
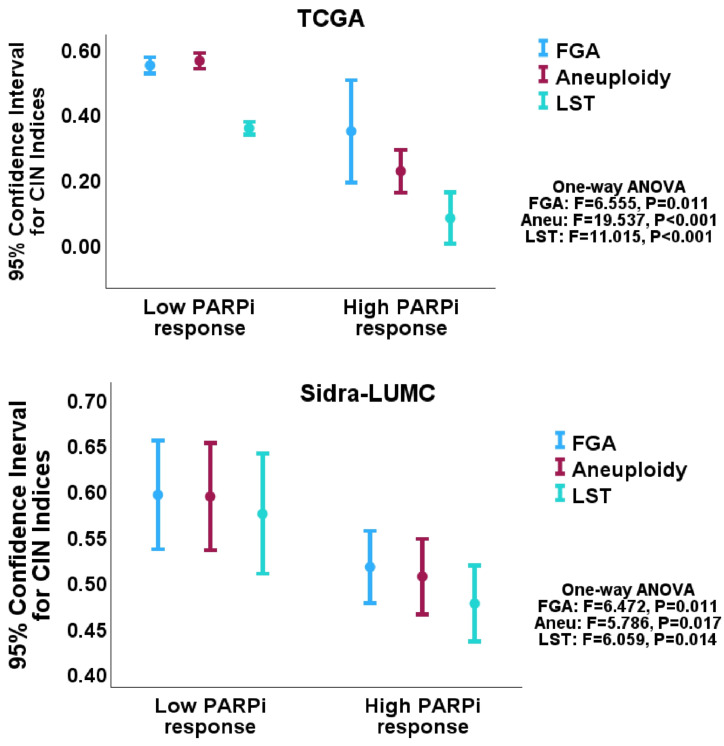
Error plots showing the inverse relationship between a PARPi response signature and the CIN indices, FGA and aneuploidy, in the TCGA (**upper panel**) and Sidra-LUMC (**lower panel**) CRC cohorts. The results confirmed the observed relationship between PARPi response and the HRD surrogate, LST. CIN = chromosomal instability; Aneu = aneuploidy; FGA = fraction genome altered; LST = large-scale state transition; HRD = homologous repair defect.

The CRC cohorts were interrogated for the identification of the predictive biomarkers of PARPi response, using GSEAs, and the compiled PARPi response gene set. The GSEA results showed that LST, MSI, *TP53* mutation status as well as *PARP1* and *PARP2* expression, but not *TP53* expression or *ATM* expression predicted an enrichment of the PARPi response gene set in both the CRC cohorts (Table 1 and Table 2). Specifically, the CRC subset with low LST, a positive MSI status, *TP53* wild-type and high *PARP1* and *PARP2* expression demonstrated enrichment for the PARPi response gene set. The CRC subsets with high LST, MSS and positive *TP53* mutations, and low *PARP1* and *PARP2* expression showed no enrichment of the PARPi response. The results showed that tumors with low CIN features (namely, low LST, *TP53* wild-type and MSI positivity) were enriched for PARPi response. To confirm whether a PARPi response was associated with low CIN in CRC, GSEA was performed using the CIN indices, FGA and Aneuploidy scores, as phenotypes. FGA- and Aneuploidy-based GSEAs confirmed the enrichment of the PARPi response in the low FGA and Aneuploidy CRC subsets. (Table 1 and Table 2; Figure 3). This is in contrast to the established relationship between HRD scores and PARPi response in breast, prostate, pancreatic and ovarian cancers.

### 2.4. PARP1 Expression, PARP2 Expression and MSI Status Exhibit Non-Inferior Associations with PARPi Response in CRC

GSEAs followed by differential enrichment score (ES) analyses were executed to compare the performance of the biomarkers *TP53* mutation, MSI status, *PARP1* expression and *PARP2* expression relative to the HRD surrogate, LST. There was no significant differential ES between LST, on the one hand, and MSI status, or *PARP1* or *PARP2* expression, on the other. The results demonstrated that MSI and *PARP1/2* expression may substitute as biomarkers of PARPi in CRC. The differential ES analysis for *TP53* mutation status demonstrated a significantly lower relationship with PARPi response in comparison to LST in the TCGA, but not in the Sidra-LUMC cohort (Table 3 and Table 4).

### 2.5. Drug Ontology Enrichment Analysis Confirms the Relative Magnitude of the Relationships Between PARPi Response Biomarkers

**Figure 4:**
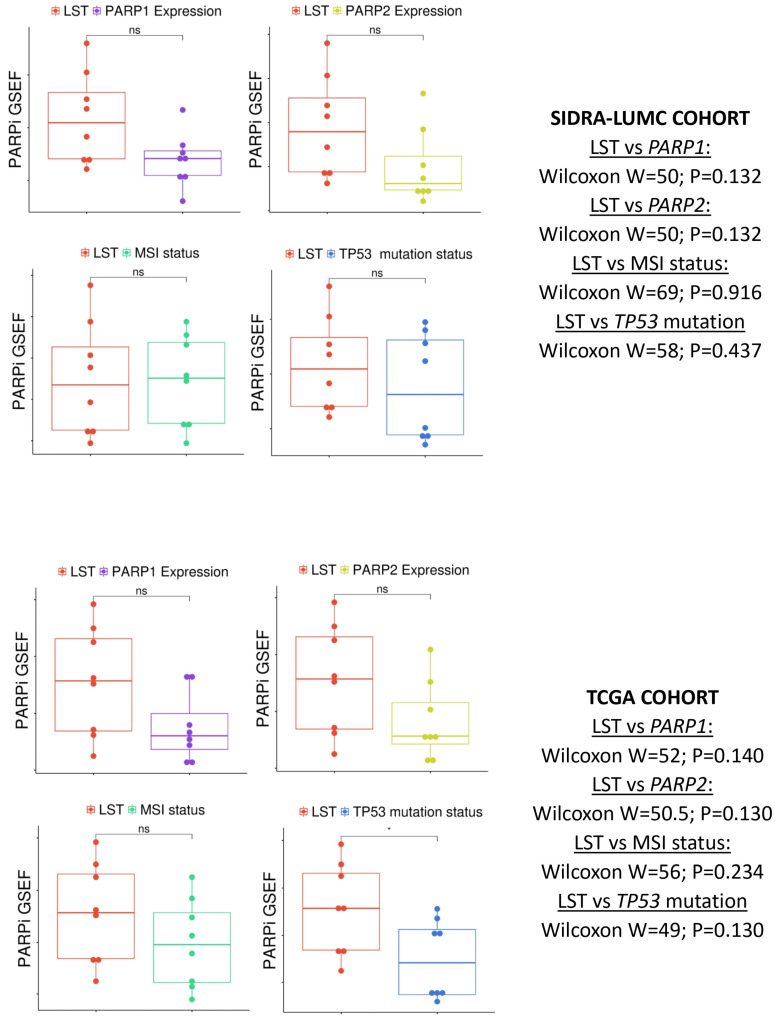
Box plots showing the GSEF analyses for the biomarkers *PARP1* expression, *PARP2* expression, MSI status and *TP53* mutation status in comparison with LST in the enrichment of PARPi response ontology terms in the Sidra-LUMC (**upper panel**) and TCGA (**lower panel**) CRC cohorts. No significant enrichment was observed between LST and each of the other biomarkers. GSEF = gene set enrichment fraction; the displayed *p* values are FDR values.

Drug ontology enrichment was performed on Enrichr for the LST-based, MSI-based, and *PARP1* and *PARP2* expression-based enrichment of PARPi gene sets using the LINCS L1000 Chem Pert Consensus Signatures. The results confirmed the association of LST, MSI, *TP53* mutation status, and *PARP1* and *PARP2* expression with PARPi response ontology terms in both CRC cohorts (Supplementary Materials S5). The Wilcox signed-rank test of the fraction of the overlapping genes between the PARPi gene modules and the biomarker-based enriched gene list (gene set enrichment fraction, GSEF) demonstrated no significant differences between LST-based enrichment and those of MSI status, and *PARP1*, and *PARP2* expression (Figure 4). Furthermore, GSEF analysis showed no differences between the LST-based enrichment and that of *TP53* wildtype-based PARPi response enrichment in either CRC cohorts.

## 3. Discussion

This study has investigated the potential utility of alternative biomarkers for PARPi response prediction in CRC. Biomarkers with known biological significance in CRC, including *TP53* mutations, MSI status and CIN markers such as FGA, aneuploidy score, in addition to the direct PARPi targets—*PARP1* and *PARP2*—were interrogated for their potential utility as PARPi response biomarkers. This study first demonstrated the clinicopathological and molecular significances of *PARP1* and *PARP2* expression in CRC, thereby laying down the basis for their interrogation as potential predictive markers for PARPi response. The interrogation of CRC biomarkers demonstrated a consistent enrichment of PARPi response for some but not all of the biomarkers in both the CRC cohorts. To the best of my knowledge, no previous study on CRC or any other cancer has interrogated *PARP1* or *PARP2* expression for their capacities as predictive biomarkers of PARPi response. More importantly, this study also showed that some of the biomarkers may have a similar performance in PARPi response prediction compared to the traditional HRD/HRD surrogates. The study findings may have implications for the clinical deployment of PARPi biomarkers, and should be of importance to clinical oncology practice in low-resource settings. Whilst *TP53* mutations, MSI and *PARP1* and *PARP2* expression testing can be performed with low throughput and more affordable molecular pathology tests such as PCR and immunohistochemistry [19,20], the assay for FGA, aneuploidy and HRD or any of its components require the deployment of high throughput and more expensive genomic technologies [8,21]. Moreover, this study may potentially have identified an additional drug group, besides immune checkpoint inhibitors [22,23], for the treatment of the MSI subset of CRC.

The patterns of PARPi enrichment observed in this study are in tandem with the results of the Smeby et al. study in two ways. First, the enrichment of the PARPi response observed for the CRC wild-type *TP53* status conforms with the Smeby et al. [8] findings. In that study, the authors demonstrated that PARP inhibition was associated with wild-type *TP53* status, even in the microsatellite stable (MSS) molecular subset of CRC. Secondly, the study demonstrated that PARPi sensitivity in CRC was not predicted by HRD-related genomic and transcriptomic signatures [8]. This is in line with the present study which showed that PARPi response was enriched in the LST-low, rather than the LST-high, subset of CRC. However, this study observed a low performance of *TP53* mutation status in predicting PARPi response enrichment, in spite of the central role *TP53* mutations play in CRC pathogenesis. Based on the findings that different *TP53* mutation types can confer differential tumor biology on cancers [24], I propose that the lumping together of all the *TP53* mutations as one subset in this study may have precluded a determination of the accurate extent of the relationship between PARPi response and *TP53* mutation status. Therefore, further research directed at the identification of PARPi response-specific *TP53* mutations in the CRC cohorts may help clarify the roles of *TP53* mutations in PARPi response and improve the performance of *TP53* mutation status in predicting PARPi response in CRC.

Whilst the findings of an association of MSI with PARPi response are in tandem with the Smeby et al. study [8], they contradict the observations of Ganther-Williams et al. (*n* = 27) [10], who demonstrated no relationship between PARPi response and MSI status. It can be argued, however, that the aforementioned contradictory study was limited by sample size. In furtherance of this argument, the sensitivity of MSI CRC to PARPi may be attributable to the loss of *MRE11* and *RAD50* that was established in the MSI CRC subset [25,26]. *MRE11* and *RAD50*, which are involved in dsDNA repair, are mutated by frameshift mechanisms in their microsatellite loci in MSI cancer subsets [26]. It is therefore conceivable that the mutation and loss of *MRE11* and *RAD50* formed a synthetic lethal mechanism with PARP inhibition in the MSI-positive CRC subset. The small sample size used in the Ganther-Williams et al. study may have precluded the power of that study to demonstrate the relationship between MSI and PARPi response. The present study utilized a sample size of over 800 samples to interrogate the relationships between the biomarkers and PARPi response in CRC.

The *PARP1* and *PARP2* biology observed for the CRC cases in this study differs significantly from their established status in breast, ovarian, prostate and pancreatic cancers. In the latter cancer types, high *PARP1* and *PARP2* expression are observed in the subsets of cancers with high chromosomal instability [27,28,29,30], hence their associations with CIN markers such as HRD, LST, TAI and LOH. These associations are related to the roles the base excision repair (BER) pathway plays in the rescue of HR-deficient cancer cells from apoptosis [31]. The BER pathway—whose members include the PARP genes—is upregulated in the breast, prostate, ovarian and prostate cancer subsets with HRD, as a secondary protective DNA repair mechanism against HRD [31]. Hence, targeting the BER pathway—specifically *PARP1* and *PARP2*—forms the basis for the “synthetic lethality” mechanism that the PARP inhibition strategy utilizes for the treatment of those cancer subsets with HRD [31]. The present study observed an association between MSI and *PARP* gene expression, although no previous study has demonstrated the specific upregulation of *PARP1* or *PARP2* in that molecular subtype of CRC, to the best of my knowledge. The association of *PARP1* and *PARP2* expression with MSI in this study explains the association of PARPi response with low LST, rather than high LST, and this demonstrates why low LST, rather than high LST, would be the predictive biomarker of PARPi response in CRC. This is in contrast with the LST-PARPi response relationship applied clinically for HRD-PARPi synthetic lethality in prostate, ovarian, breast and pancreatic cancers [31]. Furthermore, this study demonstrated that none of the high subsets of the CRC CIN markers—FGA and aneuploidy scores—were associated with PARPi response enrichment, even with their demonstrated associations with the HRD surrogate, LST. This further confirmed that PARPi response in CRC may be associated with tumors with low CIN.

LST is a suitable surrogate for HRD, and has been utilized in multiple cancer studies as a genomic signature for HRD [32,33,34]. The use of GSEA and DOEA as surrogates for PARPi response is appropriate since the enrichments of the PARPi gene sets in phenotype subsets represent changes or perturbation inducible by the PARPis [35,36]. Drug treatment can trigger changes in the expression patterns of gene clusters, and these changes in pattern are related to the mode of action of the drug, as well as to how that drug affects vital cellular processes such as apoptosis, cell cycle and cell signaling [37]. As GSEA and DOEA can be used to analyze gene expression changes, they thus represent tools that can be used to predict drug responses [38,39]. The PARPi response gene sets applied in this study were generated by the treatment of colorectal, lung, prostate, and hepatocellular cancers and leukemia cell lines with veliparib, olaparib, rucaparib and talazoparib. The genes in the sets represent those which showed significant upregulation and downregulation following treatments with PARPis [35,36]. The GSEAs and differential ES analyses results were validated using DOEAs with differential drug ontology enrichment, which is a confirmatory technique for GSEAs [40,41]. Both GSEAs and DOEAs are standard techniques for studying biological phenomena in cancers.

This study suffers from certain limitations, including (i) the fact that the gene perturbation programs utilized for PARPi response were generated from cancer cell culture studies which may not absolutely replicate the pattern of changes that may occur in natural cancers from patients, and (ii) the absence of ASCAT data for the Sidra-LUMC cohort precluded the use of HRD for a comparison of the CRC cohorts. Hence, this study is essentially a hypothesis-generating one, and a clinical translation of the identified biomarkers would require comprehensive and rigorous clinical validation. A comprehensive validation of *PARP1* and *PARP2* expression, *TP53* mutation status and MSI status as predictive biomarkers of PARPi response in CRC would require a formal, well-powered clinical trial in which PARP inhibitors are administered to CRC patients—or not—on the basis of the status of the above markers in patients’ tumor.

## 4. Materials and Methods

### 4.1. Study Approach

In this study, PARPi gene set enrichment was utilized as a surrogate for PARPi response, and enrichment scores (ESs) were used to define the relative magnitude of the relationships between biomarkers and PARPi responses. First, the relationship of PARP expression with clinicopathological, molecular and genomic indices was probed to establish a biological role for *PARP1* and *PARP2* in CRC, and interrogate the mechanisms of *PARP1* and *PARP2* deregulation in CRC. Gene set enrichment analyses (GSEAs) were performed with select gene sets for PARPi response using an HRD/HRD surrogate, FGA, aneuploidy scores, *TP53* mutation status, MSI status as well as *PARP1*, *PARP2* and *ATM* expression. Then, the predictive capacities of FGA, aneuploidy scores, *TP53* mutation status, MSI status as well as *PARP1*, *PARP2* and *ATM* expression relative to the HRD/HRD surrogate was determined using a differential ES analysis. The results of the GSEAs and differential ES analyses were confirmed with differential gene set enrichment fraction (GSEF) analysis using standard statistical tests.

### 4.2. Cancer Cohorts

This study retrospectively analyzed the clinicopathological, RNASeq and masked segment data of 537 and 348 CRC cases from the cancer genome atlas (TCGA) [42], and the Sidra-Leiden University Medical Center’s Atlas and Compass of Immune–Cancer–Microbiome (Sidra-LUMC) [43] cohorts, respectively. These data are all domiciled in the Genome Data Commons (GDC) and CBioPortal databases [44,45]. In addition, the TCGA breast cancer (BC) cohort was included in this study to validate the utility of PARPi GSEAs and DOEAs for PARPi response surrogacy, select the most sensitive PARPi response gene sets for application to the CRC cohorts, and confirm the HRD state (high versus low) with enrichment for PARPi response.

### 4.3. Data Retrieval and Processing

The clinical and genomic data of interest were extracted from the TCGA, and Sidra-LUMC data using Linux-based scripts and codes which were written in the Windows-based Ubuntu 20.04 environment. Data normalization per cohort was accomplished using fractional ranking and the method described by Templeton [46]. Following fractional ranking, the data from all three cohorts were combined and analyzed as one cohort for assessing the biological and molecular relevancies of *PARP1* and *PARP2* expression in CRC. However, for the purpose of assessing the predictive capacity of the potential PARPi response biomarkers with GSEA, the expression datasets of the three cancer cohorts were individually interrogated. This was due to the unequal records of genes in the individual datasets (TCGA = 60,483, and Sidra-LUMC = 18,355).

### 4.4. Genomic Indices

Large-scale state transitions (LST) scores, as previously defined [32], were generated from the copy number segment data (data_cng_hg19.seg), and used as a surrogate for HRD in the CRC cohorts. HRD scores, which are derived from the aggregation of large-scale state transitions (LSTs), telomeric allelic imbalance (TAI) and a loss of heterozygosity (LOH) [47], could not be generated for all three CRC cohorts because of the unavailability of ASCAT data for the Sidra-LUMC cohort. However, the HRD scores were derived for the TCGA CRC and BC cohorts from a combination of LST, LOH and TAI, and clinically relevant thresholds for the high and low scores were applied as previously described. Fraction of Genome Altered (FGA) scores were generated from the copy number segment data following the previous definitions [48]. The MSI statuses were obtained from the clinicopathological data in the TCGA and Sidra-LUMC cohorts, or derived from the other molecular subtyping schemes. The Consensus Molecular Subtype (CMS) of CRC from the Sidra-LUMC cohort was converted into two-tier (MSI versus MSS) and three-tier (Epithelial/CIN versus Hypermutated/MSI versus GS/EMT/Mesenchymal) subtyping schemes based on their described characteristics [49,50]. Tumor mutation burden (TMB) and Mutation Count were extracted from the somatic mutation data of the individual CRC cohorts.

### 4.5. Gene Set Enrichment Analysis

Gene set enrichment analysis (GSEA) was performed on the GSEA_4.3.3 software with gene sets from the Harmonizome database (https://maayanlab.cloud/Harmonizome/; accessed on 28 April 2025) [35,51]. The gene sets were derived from the cell culture studies which demonstrated the upregulation and downregulation of gene programs following treatment of the cancer cells with PARPis olaparib, rucaparib, veliparib and talazoparib [35,36]. Twenty-two gene sets from the Harmonizome database were compiled into a single gene set of 1242 genes. This compilation was prompted by the justification that all PARP inhibitors target PARP1 and PARP2, i.e., they all have the same targets; hence, they should show similar perturbation patterns. However, responses to the same PARP inhibitor may differ slightly from one cell type to another. In addition, the gene sets may have been generated under separate experimental conditions, and therefore, the perturbation patterns may show slight variations from one experiment to another. Furthermore, the 22 gene sets were generated from only a few cell lines. In recognition of these factors, the entirety of the 22 gene sets were combined into a single set to accommodate the variations that may have been induced by cellular, experimental and sample size limitations. A GSEA was first performed on the TCGA BC cohort using LOH, LST, TAI and *BRCA1* and *BRCA2* expression as the gold-standards for PARPi response biomarkers. These analyses were used to validate the PARPi gene set for PARPi response, as well as confirm the surrogacy of LST for HRD scores. Linux-based scripts were also used to prepare gct-formatted gene expression datasets as per GSEA requirements [52,53], while the phenotype and derivative gene set files were prepared in an Excel spreadsheet and converted to cls and gct files, respectively.

### 4.6. Differential Enrichment Score (ES) Analysis

To compare the strength of the association between the PARPi response biomarkers and the PARPi gene sets, a differential ES analysis was utilized to assess the preferential enrichment of the PARPi gene sets for the *PARP1/2* and *ATM* expression, *TP53* mutation and MSI statuses, or FGA in comparison to the LST-based enrichment scores. The ES values obtained for the GSEAs were input into online correlation difference calculators (http://vassarstats.net/rdiff.html; accessed on 2 May 2025; https://www.danielsoper.com/statcalc/calculator.aspx?id=104; accessed 2 May 2025) [54,55] to assess whether there were significant differences between the ES obtained for LST and those obtained for each of the other biomarkers. The basis for using a differential ES analysis is that ES is a measure of the strength of a correlation or association between a phenotype of interest and the molecular attributes or gene pathway denoted in the gene sets [52,53,56], similar to the Pearson correlation coefficient, R; hence, ES can be transformed to z-scores using Fisher z-transformation techniques [57,58].

### 4.7. Differential PARPi Response Ontology

To confirm the results of the GSEAs and differential ES analyses, drug set ontology enrichment was performed with the core-enriched genes of the olaparib, rucaparib, veliparib and talazoparib gene sets. Drug ontology enrichment was performed on the Enrichr platform using the LINCS L1000 Chem Pert Consensus Signatures. The fractions of the genes overlapping between the PARPi gene module and the biomarker-based enrichment list were compared between LST and the other individual PARPi response biomarkers using the Wilcoxon signed-rank test.

### 4.8. Statistical Analyses

The relationships among *PARP1/2* expression, clinicopathological features, molecular features and genomic features were analyzed using SPSS version 29. Fractional ranking and the normalization of continuous data were also accomplished using SPSS. The Chi square (or Fisher) test was used to probe for significant associations between categorical variables, while bivariate correlative analysis was utilized to test the correlations between continuous variables. The one-way ANOVA test was used to measure the mean differences of continuous variables between discrete groups, while the multivariate analysis was investigated with regression analyses. A *p* value of <0.05 was taken as the threshold for significant association or correlation. The Benjamini–Hochberg correction was applied for multiple testing using a false discovery rate (FDR) of 0.05. GSEA was performed with a default threshold nominal rate of 0.05 and an FDR of 0.25. The permutation number was maintained as 1000, while permutation type was set as either “gene set” or “phenotype”, as appropriate. Figure 1 and Figure 4 were produced on the SR Plots website (https://www.bioinformatics.com.cn/; accessed on 2 May 2025) [59].

## 5. Conclusions

In conclusion, this study investigated potential PARPi response biomarkers in CRC. The highlights of the investigation include a demonstration of biological relevancies for *PARP1* and *PARP2* expression in CRC; the association of low LST status with PARPi response in CRC, in contrast to the established relationship between LST and PARPi response in prostate, pancreatic, ovarian and breast cancers; and the identification of potential, alternative PARPi response biomarkers with non-inferior performances relative to LST status.

## Figures and Tables

**Table 1:** PARPi response enrichment analyses in TCGA CRC cohort.

Biomarkers	Gene Set Size	ES	NES	Nominal *p* Value	FDR q Value
LST	849	0.591	2.908	<0.001	<0.001
MSI status	849	0.550	2.582	<0.001	<0.001
*TP53* mutation status	849	0.344	1.781	<0.001	<0.001
*PARP1* Expression	849	0.665	2.438	<0.001	<0.001
*PARP2* Expression	849	0.653	2.334	<0.001	<0.001
*TP53* Expression	849	−0.262	−1.012	0.423	0.423
*ATM* Expression	849	0.172	0.512	0.982	0.982
FGA	849	0.545	2.683	<0.001	<0.001
Aneuploidy	849	0.598	2.731	<0.001	<0.001

**Table 2:** PARPi response enrichment analyses in Sidra-LUMC CRC cohort.

Biomarkers	Gene Set Size	ES	NES	Nominal *p* Value	FDR q Value
LST	667	0.419	1.595	<0.001	<0.001
MSI status	667	0.536	1.975	<0.001	<0.001
*TP53* mutation status	667	0.390	1.851	<0.001	<0.001
*PARP1* Expression	667	0.407	2.182	<0.001	<0.001
*PARP2* Expression	667	0.355	1.758	<0.001	<0.001
*TP53* Expression	667	0.384	1.541	0.053	0.053
*ATM* Expression	667	0.262	1.052	0.406	0.406
FGA	667	0.490	1.812	<0.001	<0.001
Aneuploidy	667	0.494	1.851	<0.001	<0.001

**Table 3:** Differential enrichment score analysis for the TCGA cohort.

Biomarker Pairs	N (LST vs. Other)	LST ES	Other ES	∆ ES z-Score	Adjusted *p* Value
LST vs. MSI	527 vs. 397	0.591	0.550	0.905	0.439
LST vs. *TP53* mutation status	527 vs. 491	0.591	0.343	5.103	1.98 × 10^−6^
LST vs. *PARP2* expression	527 vs. 537	0.591	0.653	−1.646	0.199
LST vs. *PARP1* expression	527 vs. 537	0.591	0.665	−1.985	0.141
LST vs. FGA	527 vs. 522	0.591	0.545	1.097	0.409
LST vs. Aneuploidy	527 vs. 524	0.591	0.598	−0.161	0.872

**Table 4:** Differential enrichment score analysis for the Sidra-LUMC cohort.

Biomarker Pairs	N (LST vs. Other)	LST ES	Other ES	∆ ES z-Score	Adjusted *p* Value
LST vs. MSI	281 vs. 348	0.419	0.536	−1.887	0.354
LST vs. *TP53* mutation status	281 vs. 281	0.419	0.390	0.297	0.858
LST vs. *PARP2* expression	281 vs. 348	0.419	0.355	0.935	0.525
LST vs. *PARP1* expression	281 vs. 348	0.419	0.407	0.180	0.858
LST vs. FGA	281 vs. 280	0.419	0.490	−1.055	0.525
LST vs. Aneuploidy	281 vs. 281	0.419	0.494	−1.118	0.525

## Data Availability

All the genomic and clinicopathological data utilized for this study are freely available in the cBioPortal for the Cancer Genomics website (https://www.cbioportal.org/, accessed on 25 April 2025), and the Genome Data Commons repository (https://portal.gdc.cancer.gov/, accessed on 25 April 2025).

## Supplementary Materials

The following supporting information can be downloaded at https://www.mdpi.com/article/10.3390/ph18060905/s1.

## Abbreviations

The following abbreviations are used in this manuscript:

TCGA	The cancer genome atlas
Sidra-LUMC	Sidra-Leiden University Medical Center’s Atlas and Compass of Immune–Cancer–Microbiome
GDC	Genome Data Commons
GSEA	Gene set enrichment analysis
DOEA	Drug ontology enrichment analysis
